# Survival After Lung Transplantation for Chronic Hypersensitivity Pneumonitis: Results From a Large International Cohort Study

**DOI:** 10.3389/ti.2022.10450

**Published:** 2022-03-31

**Authors:** Mario Nosotti, Miguel Leiva-Juarez, Frank D’Ovidio, Dirk Van Raemdonck, Laurens Ceulemans, Shaf Keshavjee, Mindaugas Rackauskas, Piero Paladini, Luca Luzzi, Paula Moreno Casado, Antonio Alvarez, Ilhan Inci, Jonas Ehrsam, Thorsten Krueger, Andrey Roth, Federico Rea, Marco Schiavon, Lorenzo Rosso

**Affiliations:** ^1^ Foundation IRCCS Cà Granda Ospedale Maggiore Policlinico, University of Milan, Milan, Italy; ^2^ Department of Surgery, Columbia University Medical Center, New York, NY, United States; ^3^ Department of Thoracic Surgery, University Hospitals Leuven, Leuven, Belgium; ^4^ Toronto Lung Transplant Program, University Health Network, Toronto, ON, Canada; ^5^ Thoracic Surgery, University Hospital of Siena (AOUS), Siena, Italy; ^6^ Division of Thoracic Surgery and Lung Transplantation, Reina Sofia University Hospital, Córdoba, Spain; ^7^ Department of Thoracic Surgery, University Hospital Zurich, Zurich, Switzerland; ^8^ Department of Surgery, Lausanne University Hospital, Lausanne, Switzerland; ^9^ Thoracic Surgery Unit, Department of Cardiologic, Thoracic and Vascular Sciences, University Hospital, Padova, Italy

**Keywords:** lung transplant, hypersensitivity pneumonitis, rare lung disease, respiratory insufficiency, pneumonia, interstitial pneumonia

## Abstract

Repeated exposure to antigens *via* inhalation is the primary cause of hypersensitivity pneumonitis, a form of interstitial pneumonia. The chronic form of hypersensitivity pneumonitis leads to progressive loss of respiratory function; lung transplantation is the only therapeutic option for chronically ill patients. The ESTS Lung Transplantation Working Group conducted a retrospective multicentred cohort study to increase the body of knowledge available on this rare indication for lung transplantation. Data were collected for every patient who underwent lung transplant for hypersensitivity pneumonitis in participating centres between December 1996 and October 2019. Primary outcome was overall survival; secondary outcome was freedom from chronic lung allograft dysfunction. A total of 114 patients were enrolled from 9 centres. Almost 90% of patients were diagnosed with hypersensitivity pneumonitis before transplantation, yet the antigen responsible for the infection was identified in only 25% of cases. Eighty per cent of the recipients received induction therapy. Survival at 1, 3, and 5 years was 85%, 75%, and 70%, respectively. 85% of the patients who survived 90 days after transplantation were free from chronic lung allograft dysfunction after 3 years. The given study presents a large cohort of HP patients who underwent lung transplants. Overall survival rate is higher in transplanted hypersensitivity pneumonitis patients than in those suffering from any other interstitial lung diseases. Hypersensitivity pneumonitis patients are good candidates for lung transplantation.

## Graphical Abstract

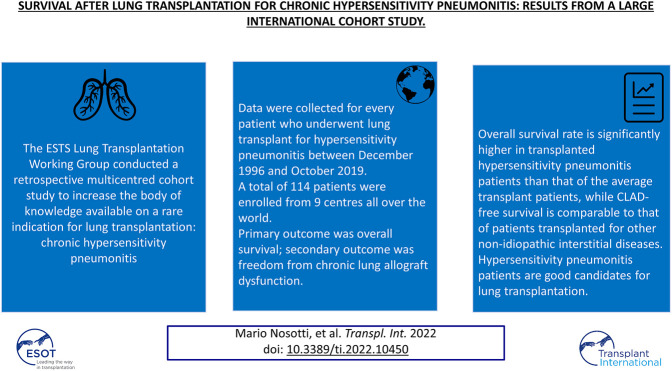

## Introduction

Hypersensitivity pneumonitis (HP) is a rare parenchymal disease prompted by an immunologic reaction to inhaled organic antigens. HP incidence was assessed as 1 per 100,000 inhabitants in Great Britain; its prevalence varies significantly among countries, regions, and according to occupational exposure (1). A study based on insurance claims databases conducted in the United States between 2004 and 2013 estimated the prevalence of HP to be from 1.67 to 2.71 per 100,000 inhabitants (2).

Repeated exposure to one or more stimulating agents triggers the onset of HP in susceptible individuals; these patients develop both a humoral and a cellular reaction, which leads to peribronchiolar chronic inflammatory infiltrates and non-necrotising granulomas. The presence of MUC5B (Mucin 5B) single nucleotide polymorphisms and peripheral blood leukocyte telomere length dysfunction seems to induce pulmonary fibrosis in HP patients (3). The development of fibrosis can follow three different patterns: simple peribronchiolar, subpleural or bridging fibrosis. The latter is quite typical of HP, because of which spreading fibrotic tissue in the interstitium between bronchioles and interlobular septa or areas of subpleural fibrosis accumulates (4).

It is well known that the diagnosis of HP is not straightforward. A 2016 study, conducted on 70 England-based patients affected with interstitial pulmonary disease, assessed the difficulties encountered by international multidisciplinary teams in reaching a consensus on diagnosis and therapeutic approach (5). Very recently, an *ad hoc* board of experts appointed by several scientific societies drew up a practical clinical guideline for diagnosing HP in adults (6). The guideline board developed a systematic approach to diagnostic criteria and established a dedicated algorithm based on computer tomography imaging, exposure evaluation, broncho-alveolar lavage lymphocytosis, and histopathological findings. The board drew a definite distinction between the fibrotic and non-fibrotic forms of HP; the first is clearly associated with a poor prognosis. Indeed, findings of thick fibrosis, fibroblast foci, and microscopic honeycombing in a cohort of 119 patients with HP were predictors of early mortality or lung transplantation (7).

Patients with HP who develop the fibrotic form of HP can generally benefit from lung transplantation; nevertheless, the opportunity to enlist patients who have shown an extreme pathological reaction to a foreign antigen and would be permanently exposed to a graft only partially compatible with their immune system raises some concerns. Being HP a rare clinical occurrence, the scientific literature is lacking dedicated studies on the topic. The European Society of Thoracic Surgeons Lung Transplantation Working Group (ESTS-LTxWG) on lung transplantation deemed necessary to help fill this gap in the literature with a large multicentred retrospective study, given the consistent number of patients with HP enrolled in a previous ESTS-LTxWG study on rare indications for lung transplantation (8).

## Materials and Methods

This was an international, retrospective cohort study including consecutive patients who received lung transplantation in 9 centres between Europe and North America. Each centre autonomously identified suitable patients and collected the data. Eligible patients were adult individuals with histologically proven HP on native lungs; each centre was responsible for the proper diagnosis of their own patients. Postoperative therapy, as well as periodical clinical assessments, followed the standard of care in each participating centre. Demographic, surgical, and survival data were collected with standardised database templates to warrant reliable data collection.

The primary objective of the current study was to evaluate the effects of lung transplantation in patients with HP in terms of overall survival, which was calculated from the day of lung transplantation until death or last follow-up. Secondary outcome was chronic lung allograft dysfunction (CLAD). The onset of CLAD in each patient was diagnosed individually according to the consensus report from the Pulmonary Council of the International Society for Heart and Lung Transplantation (ISHLT) (9). The authors also analysed the outcome of single versus double transplantation and induction therapy versus no induction therapy.

### Statistical Analysis

Continuous data were presented as mean and standard deviation or median and 1st to 3rd quartile. Categorical variables are shown as absolute and percentage frequencies. Time-to-event data were displayed using non-parametric Kaplan Meier estimators. The hazard ratio (HR) was computed using Cox regression models with Breslow approximation; given the multicentric nature of collected data, a robust sandwich variance estimator was adopted to account for correlated groups of observations. The CLAD variable was treated as a time-varying covariate into Cox models. The proportional hazards assumption was checked using statistical tests and graphical diagnostics based on the scaled Schoenfeld residuals. Confidence intervals were computed at 95%, and side p-values were considered significant when < 0.05. All analyses were carried out using R-Cran software, version 3.5.3.

This study followed the principles outlined in the Declaration of Helsinki (2013), was approved by the Institutional Review Board (749_2016bis; Milan 2) and received no financial support.

## Results

One-hundred and fourteen patients were eligible and enrolled from seven European and two North American transplantation centres. Participating centres and the respective number of recruited patients are listed in Supplementary Table S1. Main clinical characteristics in patient cohort receiving lung transplantation between December 1996 and October 2019 are summarised in Table 1. The patient cohort showed a slight prevalence of female patients and a median age of 57 years. The 89.5% of patients were diagnosed with HP prior to listing. However, the antigen responsible for the onset of their lung disease could be identified in only 25.4% of cases. The vast majority of patients in whom the antigen could be identified had been exposed to birds (72.4%). Patients were treated with bilateral transplantation in 45.2% of cases. Induction immunosuppressive therapy rate was 80%. More than 84% of patients were transplanted in the last decade. Median follow-up was 2.25 years.

**TABLE 1:** Patients’ characteristics.

Variable	Value
Number	114
Male gender	71 (62.3%)
Age, years	57.5 (50–63)
Preoperative diagnosis	102 (89.5%)
Exposure to antigens	
Bird fanciers	21 (18.4%)
Farmers, mushroom growers, gardeners	5 (4.4%)
Pharmaceutical industry workers	3 (2.6%)
Not known	85 (74.6)
Preoperative FEV1%	41.3 (32–54)
Preoperative FVC%	40 (33–51.7)
Preoperative DLCO%^a^	32 (23–38)
Bilateral transplantation	52 (45.6%)
Transplantations by era (2009–2019)	96 (84.2%)
Induction therapy	91 (79.8%)

Data are presented as number and percentage or median and 1st to 3rd quartile; FEV1%: percentage of predicted forced expiratory volume in 1 s; FVC%: percentage of predicted forced vital capacity; DLCO%: percentage of predicted diffusion capacity CO.

a Data from 38 patients only.

**FIGURE 1:**
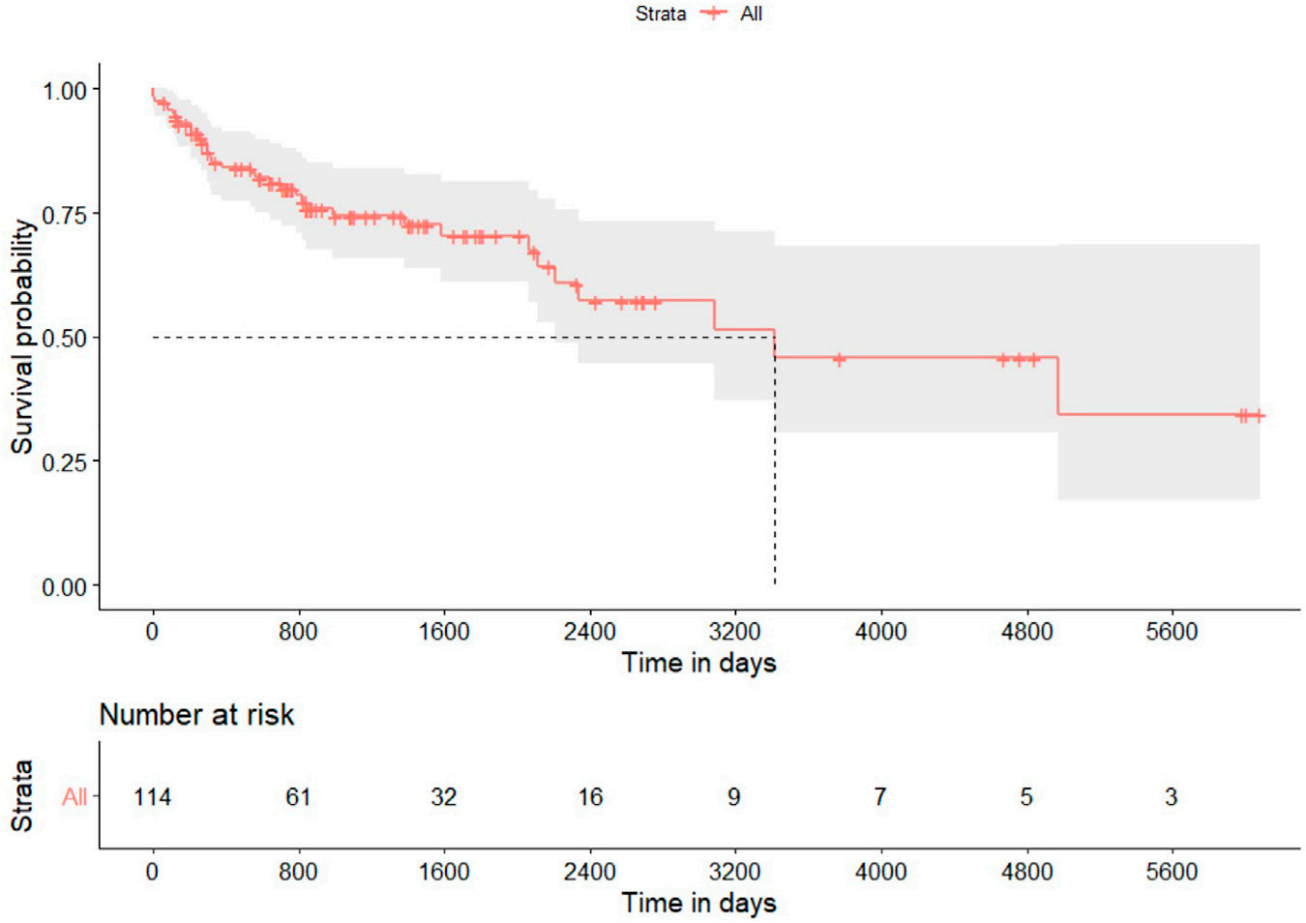
Kaplan-Meier plot for overall survival after lung transplantation for hypersensitivity pneumonitis. Gray area identifies the 95% pointwise confidence intervals.

Five (4.4%) patients died within 90 days of transplantation: three patients experienced cardiovascular events, one had hyperacute rejection, and one suffered from a surgical complication. Figure 1 shows the Kaplan-Meier plot for overall survival. Survival rate at 1, 3 and 5 years was 85.2%, 74.4% and 70.4%, respectively; median survival was 9.2 years (Table 2). Multivariable Cox proportional hazard regression model adjusted for age showed a higher hazard of death over time for patients with CLAD (HR = 9.10; 95% CI from 6.10 to 13.53; *p* < 0.001) and a lower risk of death for patients treated with induction therapy (HR = 0.45; 95% CI from 0.23 to 0.87; p 0.017). Giving that the variable mono/bilateral transplantation violated the Cox proportional hazard assumption, analyses were repeated on each subgroup. Multivariable Cox model adjusted for age showed that patients with single lung transplantation who developed CLAD experienced a higher hazard of mortality compared with patients without CLAD (HR = 8.86; 95% CI from 4.59 to 17.8; *p* < 0.001). Conversely, a refitted Cox model adjusted for age showed that patients with single lung transplantation had a lower risk of mortality if treated with induction therapy (HR = 0.26; 95% CI from 0.11 to 0.61; *p* < 0.002). Cox model adjusted for age showed in the bilateral transplantation group an HR of death for CLAD of 9.74 (95%CI from 7.04 to 13.45; *p* < 0.001); the refitted model adjusted for age indicated a not significant reduction hazard of death related to induction therapy (HR = 0.52; 95% CI from 0.15 to 1.75; *p* = 0.289).

**TABLE 2:** Survival outcomes.

Variable	Value
Mortality at 90 days	5 (4.4%)
Overall survival	
Events (mortality)	34 (29.8%)
Median survival (years)	9.2
1-year survival rate	85.2% (from 78.7% to 92.2%)
3-year survival rate	74.4% (from 66.0% to 83.8%)
5-year survival rate	70.4% (from 61.0% to 81.1%)

Data are presented as number and percentage, median or rate and 95% confidence interval.

**FIGURE 2:**
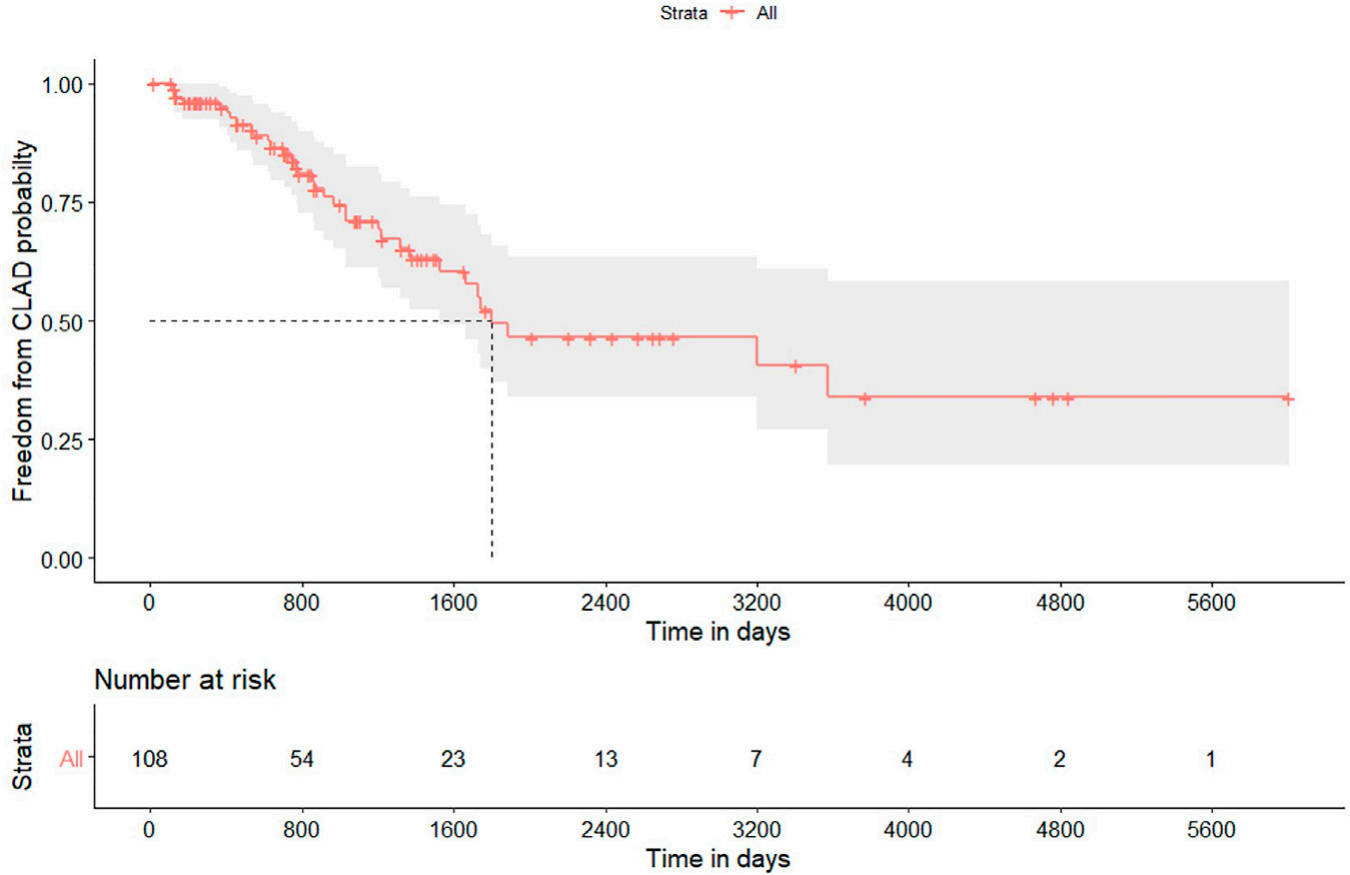
Kaplan-Meier plot for freedom to chronic lung allograft dysfunction in patients who survived 90 days after lung transplantation for hypersensitivity pneumonitis. Gray area identifies the 95% pointwise confidence intervals.

Thirty-four out of 108 patients who survived beyond 90 days after transplantation developed CLAD; Table 3 reports some characteristics, while Figure 2 displays the freedom from CLAD Kaplan-Meier graph. CLAD-free survival at 1, 3, and 5 years was 95%, 71% and 49.3%, respectively. Univariable Cox analysis for CLAD identified induction therapy in the single lung transplantation subgroup (HR = 0.48; 95% CI from 0.25 to 0.91; p 0.025) as a protective factor; induction therapy after bilateral transplantation did not reach the statistical significance in the refitted univariable model (HR = 0.62; 95% CI from 0.24 to 1.63; p 0.339).

**TABLE 3:** Chronic lung allograft disease in patients who survived 90 days after transplantation.

Variable	Value
Number	108
Male gender	67 (62.0%)
Age	58 (51–63)
Bilateral transplantation	49 (45.4%)
Induction therapy	87 (80.6%)
Median follow-up, days	810 (399–1440)
Patients with diagnosis of CLAD	34 (31.5%)
Median CLAD-free survival, days	1800
CLAD-free survival	
1-year survival (95%CI)	95.0% (from 91.0% to 99.4%)
3-year survival (95%CI)	71.0% (from 61.2% to 82.4%)
5-year survival (95%CI)	49.3% (from 37.9% to 65.8%)

Data are presented as number and percentage or median and 1st to 3rd quartile; CLAD: chronic lung allograft dysfunction.

## Discussion

Lung transplantation for HP is infrequent; this condition is usually included in the extensive list of interstitial lung diseases (excluding idiopathic interstitial pneumonia), which only make up 5.7% of all lung transplantations, according to the 2019 Thoracic Organ Transplant Registry report of the International Society for Heart and Lung Transplantation (10). Being HP a rare disease, reports of outcomes after lung transplantation are scarce. To the best of the authors’ knowledge, only one paper drafted by Kern and collaborators specifically addressed this issue (11). The researchers compared 31 patients with HP to 91 patients with idiopathic pulmonary fibrosis; patients’ survival at 1, 3, and 5 years after transplantation in HP patients was 96%, 89%, and 89%, respectively. Survival rates among HP patients were far higher than those recorded in the idiopathic pulmonary fibrosis cohort. Moreover, a reduced rate of acute cellular rejection was observed in the first year after transplantation in patients with HP. Finally, the authors found two possible HP recurrences after transplantation.

Although the Californian study was excellent in methodology and interpretation, it suffered from the typical lack of external validation of monocentric studies; therefore, some form of multicentric validation would have been advisable. This international collaborative study confirmed the excellent overall survival fixing median survival after lung transplantation for HP at 9.2 years. This result is particularly encouraging in light of the fact that the group of pathologies in which HP is included reaches a median survival of 6.4 years, according to the ISHLT TTX report (10).

Despite survival in this cohort being equivalent either after single or bilateral lung transplantation, the violation of proportional hazard assumption verified through the Schoenfeld residuals test prevented us from performing proper multivariable analyses on the entire patient group. Therefore, by dividing the cohort by transplant type, it has been proved that the CLAD onset had a strong negative impact on survival in both subgroups (HR 8.86 and 9.74 for single and bilateral lung transplantation, respectively). Notwithstanding the excellent survival of patients transplanted for HP, negative effects of chronic rejection were also observed in this cohort (12). Induction therapy impacted positively on survival in the subgroup of patients treated with single lung transplantation. This result is likely linked to the small sample size, given that it has been already shown elsewhere how this variable is protective for both types of lung transplantation (8).

Median CLAD-free survival in our cohort was 4.9 years; this result was satisfactory and congruent with the time span (4.8 years) recorded in the ISHLT TTX report concerning patients transplanted for interstitial lung diseases excluding idiopathic interstitial pneumonia (10). Considering how this patient cohort CLAD included the possible recurrence of HP in addition to bronchiolitis obliterans syndrome and restrictive allograft syndrome, one can speculate that the recurrence of underlying lung disease had a negligible clinical impact. The given study found that induction therapy is likely to have a protective effect against the onset of CLAD only in patients who underwent single lung transplantation.

The current study has some limitations. As a multicentric retrospective study, it is susceptible to selection bias; namely, we have not been able to classify patients according to recent HP guidelines (6) since our data collection ended before their publication. Given the radiological and pathological peculiarities of HP, it is unlikely that incorrect diagnoses were made for enrolled patients, while some cases may have been classified as idiopathic interstitial pneumonia and therefore not included in the current study. Another limitation is the absence of a control group. We took as reference the ISHLT Thoracic Organ Transplant Registry data for patients suffering from idiopathic fibrosis. Anyway, it cannot be entirely excluded that the results obtained by the centres participating in the study were, for some reason, above the international average limiting the difference in survival with HP patients. Moreover, the chance that unknown clinical factors may have affected the observed results cannot be ruled out. In particular, no data on possible recurrence of HP in the graft are available. Among the patients with CLAD the prevalence of the restrictive form was 11.7%; this prevalence is lower than that reported in the literature for the general population of patients transplanted with CLAD. We can speculate that HP recurrence, which has a clinical picture similar to the restrictive allograft syndrome, had a negligible impact on our patient cohort. Another limitation is the high percentage of patients in whom the antigen was not known; theoretically, different antigens could affect the aggressiveness of pulmonary fibrosis and therefore determine different underlying clinical conditions. One can speculate that this possible confounder, which is geographically determined, was mitigated by the international distribution of this patient cohort.

In conclusion, this international multicentric study highlights how patients with HP are good candidates for lung transplantation. Their survival rate is significantly higher than that of the average transplant patients, while CLAD-free survival is comparable to that of patients transplanted for other non-idiopathic interstitial diseases. The problem of possible recurrence of HP in the graft requires additional studies, although its clinical impact seems very limited.

## Data Availability

The original contributions presented in the study are included in the article/Supplementary Material, further inquiries can be directed to the corresponding author.

## Abbreviations

CI, confidence interval; CLAD, chronic-lung-allograft-dysfunction; ESTS-LTxWG, European society of thoracic surgeons lung transplantation Working Group; HP, hypersensitivity pneumonitis; HR, hazard ratio; ISHLT, International society for heart and lung transplantation.
