# Supplementary material for: Survival After Lung Transplantation for Chronic Hypersensitivity Pneumonitis: Results From a Large International Cohort Study
Source: Transpl Int. 2022 Mar 31;35:10450. doi: 10.3389/ti.2022.10450 (PMC9008138; doi:10.3389/ti.2022.10450)
Supplement: Supplementary file 1 [file Table1.docx]

Table S1. Recruiting centers and number of patients.

| **Transplantation center and location** | **Patients** | **Rate on total transplantation procedures** |
| --- | --- | --- |
| Columbia University Medical Center, New York, United States | 58 | 3.6% |
| University Hospitals, Leuven, Belgium | 25 | 2.3% |
| University of Toronto, Toronto, Canada | 11 | 0.7% |
| University of Siena, Siena, Italy | 6 | 3.1% |
| University Hospital Reina Sofia, Cordoba, Spain | 4 | 1.6% |
| University of Milan, Milan, Italy | 4 | 1.3% |
| Zurich University Hospital, Zurich, Switzerland | 4 | 0.8% |
| Lausanne University Hospital, Lausanne, Switzerland | 1 | 0.3% |
| Padua University Hospital, Padua, Italy | 1 | 0.2% |
